# Sodium 5-amino-1,3,4-thia­diazole-2-thiol­ate dihydrate

**DOI:** 10.1107/S1600536809051897

**Published:** 2009-12-04

**Authors:** Jiayi Wu, Yifeng Wang, Guo Yi, Shuping Luo

**Affiliations:** a State Key Laboratory Breeding Base of Green Chemistry-Synthesis Technology, Zhejiang University of Technology, Hangzhou, 310014, People’s Republic of China

## Abstract

There are two 5-amino-1,3,4-thia­diazole-2(3*H*)-thiolate anions in the asymmetric unit of the title compound, Na^+^·C_2_H_2_N_3_S_2_^−^·2H_2_O, which are almost perpendicular to each other [dihedral angle = 84.64 (6)°]. The two Na^+^ cations are in distorted fourfold coordinations by O atoms of the water molecules. The crystal structure is stabilized by N—H⋯S, O—H⋯N and O—H⋯S hydrogen bonds.

## Related literature

For use of 5-amino-1,3,4-thia­diazole-2(3〈i〉H〈/i〉)-thione deriv­atives as inter­mediates for pharmaceuticals, see: John & Gilmer (1960 ▶); John (1962 ▶); For related structures, see: Downie *et al.* (1971 ▶); Deng *et al.* (2005 ▶); Ma *et al.* (2007 ▶).
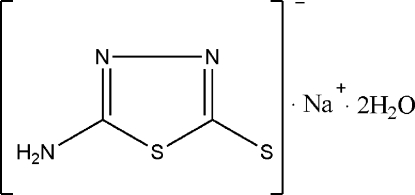


## Experimental

### 

#### Crystal data

Na^+^·C_2_H_2_N_3_S_2_^−^·2H_2_O
*M* *_r_* = 191.21
Monoclinic, 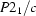

*a* = 8.7810 (3) Å
*b* = 20.0593 (5) Å
*c* = 8.4351 (3) Å
β = 91.026 (1)°
*V* = 1485.53 (8) Å^3^
*Z* = 8
Mo *K*α radiation
μ = 0.72 mm^−1^
*T* = 296 K
0.38 × 0.28 × 0.17 mm

#### Data collection

Rigaku R-AXIS RAPID diffractometer
Absorption correction: multi-scan (*ABSCOR*; Higashi, 1995 ▶) *T*_min_ = 0.758, *T*_max_ = 0.885
14264 measured reflections
3376 independent reflections
2974 reflections with *I* > 2σ(*I*)
*R*_int_ = 0.026

#### Refinement

*R*[*F* ^2^ > 2σ(*F* ^2^)] = 0.026
*wR*(*F* ^2^) = 0.059
*S* = 1.00
3376 reflections
182 parameters
H-atom parameters constrained
Δρ_max_ = 0.30 e Å^−3^
Δρ_min_ = −0.32 e Å^−3^

### 

Data collection: *PROCESS-AUTO* (Rigaku/MSC, 2006 ▶); cell refinement: *PROCESS-AUTO*; data reduction: *CrystalStructure* (Rigaku/MSC, 2007 ▶); program(s) used to solve structure: *SHELXS97* (Sheldrick, 2008 ▶); program(s) used to refine structure: *SHELXL97* (Sheldrick, 2008 ▶); molecular graphics: *ORTEP-3 for Windows* (Farrugia, 1997 ▶); software used to prepare material for publication: *WinGX* (Farrugia, 1999 ▶).

## Supplementary Material

Crystal structure: contains datablocks global, I. DOI: 10.1107/S1600536809051897/pk2208sup1.cif

Structure factors: contains datablocks I. DOI: 10.1107/S1600536809051897/pk2208Isup2.hkl

Additional supplementary materials:  crystallographic information; 3D view; checkCIF report

## Figures and Tables

**Table 1:** Hydrogen-bond geometry (Å, °)

*D*—H⋯*A*	*D*—H	H⋯*A*	*D*⋯*A*	*D*—H⋯*A*
O2*A*—H104⋯N3*B*	0.85	1.99	2.8113 (18)	161
O1*A*—H102⋯S1*B*	0.87	2.39	3.2563 (14)	172
O2*B*—H201⋯S1*B*^i^	0.86	2.45	3.2962 (12)	169
O2*B*—H202⋯N2*B*^ii^	0.86	1.95	2.8024 (18)	170
N1*A*—H1*A*2⋯S1*B*^iii^	0.86	2.57	3.4081 (16)	165
N1*B*—H1*B*2⋯S1*A*^iv^	0.86	2.43	3.2589 (17)	161

Symmetry codes: (i) 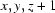; (ii) 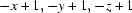; (iii) 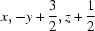; (iv) 
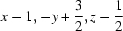
.

**Fig. 1.**
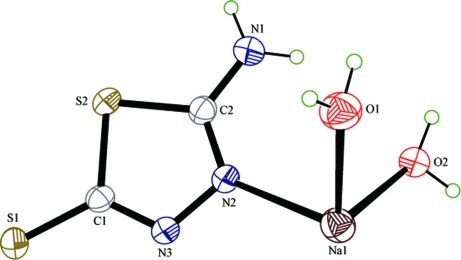
The asymmetric unit of the title compound with the atomic labeling scheme. Displacement ellipsoids are drawn at the 40% probability level.

**Fig. 2.**
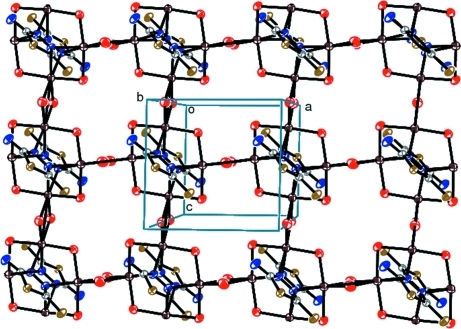
A partial packing diagram of title compound.

## supplementary crystallographic information

### Comment

Interest in the study of 5-amino-1,3,4-thiadiazole-2(3*H*)-thione derivatives stems from their use as intermediates of pharmaceuticals (John *et al.*, 1960; John, 1962).  Nonetheless, there are few articles that describe this kind of crystal structure (Downie *et al.*, 1972; Deng *et al.*, 2005; Ma *et al.*, 2007). As part of our studies of agrochemicals, the title compound 5-amino-2-thione-1,3,4-thiadiazole sodium dihydrate has been synthesized, and its crystal structure is reported in this article. The complex is located across an inversion centre, and is bridged by two symmetry equivalent water molecules Na—O (bridge) distances of 2.3776 (14) Å and 2.5141 (15) Å and an Na—O—Na bond angle of 98.99 (3)°. It has two 5-amino-1,3,4-thiadiazole-2(3*H*)-thione molecules in the asymmetric unit that are almost perpendicular to each other [dihedral angle = 84.64 (6)°]. The structure is stabilized by N—H···S, O—H···N and O—H···S hydrogen bonds.

### Experimental

5-amino-1,3,4-thiadiazole-2(3*H*)-thione(0.1 mmol) and sodium hydroxide (0.1 mmol) were dissolved in water (10 ml) and stirred for 3 h. The water was then removed under reduced pressure. Single crystals were obtained by slow evaporation of a methanol solution at room temperature.

### Refinement

All H atoms were initially located in a difference Fourier map. N-bound H atoms were located in a difference map and refined with an N—H distance restraint of 0.86 (1) Å. The water H atoms were refined using a riding model, with *U*_iso_(H)=1.5_eq_(O).

### Crystal data

**Table:**

Na^+^·C_2_H_2_N_3_S_2_^−^·2H_2_O	*F*(000) = 784
*M**_r_* = 191.21	*D*_x_ = 1.710 Mg m^−^^3^
Monoclinic, *P*2_1_/*c*	Mo *K*α radiation, λ = 0.71073 Å
Hall symbol: -P 2ybc	Cell parameters from 11555 reflections
*a* = 8.7810 (3) Å	θ = 3.0–27.4°
*b* = 20.0593 (5) Å	µ = 0.72 mm^−^^1^
*c* = 8.4351 (3) Å	*T* = 296 K
β = 91.026 (1)°	Block, colorless
*V* = 1485.53 (8) Å^3^	0.38 × 0.28 × 0.17 mm
*Z* = 8	

### Data collection

**Table:**

Rigaku R-AXIS RAPID diffractometer	3376 independent reflections
Radiation source: rolling anode	2974 reflections with *I* > 2σ(*I*)
graphite	*R*_int_ = 0.026
Detector resolution: 10.00 pixels mm^-1^	θ_max_ = 27.4°, θ_min_ = 3.1°
ω scans	*h* = −11→11
Absorption correction: multi-scan (*ABSCOR*; Higashi, 1995)	*k* = −25→25
*T*_min_ = 0.758, *T*_max_ = 0.885	*l* = −10→10
14264 measured reflections	

### Refinement

**Table:**

Refinement on *F*^2^	Secondary atom site location: difference Fourier map
Least-squares matrix: full	Hydrogen site location: inferred from neighbouring sites
*R*[*F*^2^ > 2σ(*F*^2^)] = 0.026	H-atom parameters constrained
*wR*(*F*^2^) = 0.059	*w* = 1/[σ^2^(*F*_o_^2^) + (0.014*P*)^2^ + 1.*P*] where *P* = (*F*_o_^2^ + 2*F*_c_^2^)/3
*S* = 1.00	(Δ/σ)_max_ = 0.001
3376 reflections	Δρ_max_ = 0.30 e Å^−^^3^
182 parameters	Δρ_min_ = −0.32 e Å^−^^3^
0 restraints	Extinction correction: *SHELXL*, Fc^*^=kFc[1+0.001xFc^2^λ^3^/sin(2θ)]^-1/4^
Primary atom site location: structure-invariant direct methods	Extinction coefficient: 0.0516 (12)

### Special details

**Table:**

Geometry. All e.s.d.'s (except the e.s.d. in the dihedral angle between two l.s. planes) are estimated using the full covariance matrix. The cell e.s.d.'s are taken into account individually in the estimation of e.s.d.'s in distances, angles and torsion angles; correlations between e.s.d.'s in cell parameters are only used when they are defined by crystal symmetry. An approximate (isotropic) treatment of cell e.s.d.'s is used for estimating e.s.d.'s involving l.s. planes.
Refinement. Refinement of *F*^2^ against ALL reflections. The weighted *R*-factor *wR* and goodness of fit *S* are based on *F*^2^, conventional *R*-factors *R* are based on *F*, with *F* set to zero for negative *F*^2^. The threshold expression of *F*^2^ > 2σ(*F*^2^) is used only for calculating *R*-factors(gt) *etc*. and is not relevant to the choice of reflections for refinement. *R*-factors based on *F*^2^ are statistically about twice as large as those based on *F*, and *R*- factors based on ALL data will be even larger.

### Fractional atomic coordinates and isotropic or equivalent isotropic displacement parameters (Å^2^)

**Table:**

	*x*	*y*	*z*	*U*_iso_*/*U*_eq_	
S2B	0.46866 (5)	0.720405 (18)	0.04494 (5)	0.03114 (11)	
S2A	0.97675 (5)	0.708164 (18)	0.53837 (5)	0.03013 (10)	
S1B	0.71560 (5)	0.66512 (2)	−0.16488 (6)	0.03952 (12)	
S1A	1.23359 (5)	0.65778 (2)	0.75020 (5)	0.03794 (12)	
Na1B	0.71086 (8)	0.49937 (4)	0.47238 (8)	0.04359 (19)	
Na1A	0.98033 (7)	0.50450 (3)	0.20940 (8)	0.03560 (16)	
O2A	0.70915 (13)	0.49947 (6)	0.18979 (14)	0.0359 (3)	
H104	0.6554	0.5221	0.1237	0.054*	
H103	0.6897	0.4585	0.1699	0.054*	
O1A	0.99706 (15)	0.58368 (6)	0.00068 (15)	0.0446 (3)	
H101	1.0720	0.6080	−0.0267	0.067*	
H102	0.9227	0.6081	−0.0358	0.067*	
O2B	0.75550 (13)	0.50610 (6)	0.74664 (14)	0.0347 (3)	
H204	0.5182	0.6062	0.4294	0.052*	
H203	0.5156	0.6066	0.5812	0.052*	
N2B	0.41175 (17)	0.60144 (7)	0.13535 (18)	0.0379 (3)	
N2A	0.93910 (16)	0.58939 (6)	0.43221 (16)	0.0320 (3)	
N3B	0.53241 (16)	0.59605 (7)	0.03004 (17)	0.0356 (3)	
O1B	0.50110 (17)	0.57964 (7)	0.50136 (16)	0.0540 (4)	
H201	0.7328	0.5460	0.7754	0.081*	
H202	0.6964	0.4767	0.7880	0.081*	
N3A	1.05908 (15)	0.58553 (6)	0.54520 (15)	0.0307 (3)	
C2A	0.88628 (18)	0.65001 (7)	0.41696 (18)	0.0278 (3)	
N1A	0.76956 (18)	0.66682 (7)	0.31696 (18)	0.0426 (4)	
H1A1	0.7261	0.6368	0.2592	0.051*	
H1A2	0.7391	0.7075	0.3114	0.051*	
C1B	0.57417 (17)	0.65311 (7)	−0.02914 (18)	0.0277 (3)	
C2B	0.36620 (18)	0.66293 (8)	0.15350 (18)	0.0304 (3)	
N1B	0.24845 (18)	0.67989 (8)	0.2467 (2)	0.0479 (4)	
H1B1	0.2000	0.6496	0.2972	0.057*	
H1B2	0.2221	0.7210	0.2554	0.057*	
C1A	1.09218 (17)	0.64295 (7)	0.61080 (17)	0.0262 (3)	

### Atomic displacement parameters (Å^2^)

**Table:**

	*U*^11^	*U*^22^	*U*^33^	*U*^12^	*U*^13^	*U*^23^
S2B	0.0345 (2)	0.02059 (18)	0.0384 (2)	−0.00051 (15)	0.00191 (16)	0.00096 (15)
S2A	0.0354 (2)	0.02064 (18)	0.0342 (2)	0.00192 (15)	−0.00284 (16)	−0.00291 (15)
S1B	0.0396 (2)	0.0309 (2)	0.0485 (3)	0.00355 (17)	0.01226 (19)	0.00579 (18)
S1A	0.0416 (2)	0.0306 (2)	0.0411 (2)	−0.00624 (17)	−0.01399 (18)	0.00242 (17)
Na1B	0.0385 (4)	0.0584 (5)	0.0339 (4)	0.0037 (3)	0.0013 (3)	0.0044 (3)
Na1A	0.0322 (3)	0.0395 (4)	0.0351 (3)	0.0030 (3)	0.0002 (3)	−0.0019 (3)
O2A	0.0390 (6)	0.0304 (6)	0.0380 (6)	0.0020 (5)	−0.0086 (5)	−0.0004 (5)
O1A	0.0437 (7)	0.0404 (7)	0.0497 (7)	−0.0024 (6)	0.0003 (6)	0.0079 (6)
O2B	0.0352 (6)	0.0321 (6)	0.0370 (6)	−0.0011 (5)	0.0036 (5)	0.0002 (5)
N2B	0.0408 (8)	0.0264 (7)	0.0467 (8)	0.0025 (6)	0.0098 (6)	0.0080 (6)
N2A	0.0395 (7)	0.0238 (6)	0.0323 (7)	0.0009 (6)	−0.0083 (6)	−0.0022 (5)
N3B	0.0383 (8)	0.0251 (7)	0.0437 (8)	0.0045 (6)	0.0069 (6)	0.0060 (6)
O1B	0.0642 (9)	0.0502 (8)	0.0478 (8)	0.0032 (7)	0.0073 (7)	0.0103 (6)
N3A	0.0358 (7)	0.0230 (6)	0.0330 (7)	0.0014 (5)	−0.0071 (6)	−0.0008 (5)
C2A	0.0318 (8)	0.0251 (7)	0.0266 (7)	−0.0002 (6)	−0.0001 (6)	−0.0010 (6)
N1A	0.0486 (9)	0.0313 (7)	0.0473 (9)	0.0050 (6)	−0.0182 (7)	−0.0007 (6)
C1B	0.0284 (7)	0.0240 (7)	0.0304 (7)	0.0020 (6)	−0.0050 (6)	0.0014 (6)
C2B	0.0325 (8)	0.0278 (8)	0.0308 (8)	−0.0020 (6)	−0.0017 (6)	0.0029 (6)
N1B	0.0521 (10)	0.0336 (8)	0.0588 (10)	0.0017 (7)	0.0236 (8)	0.0014 (7)
C1A	0.0296 (7)	0.0233 (7)	0.0257 (7)	−0.0010 (6)	0.0020 (6)	0.0029 (6)

### Geometric parameters (Å, °)

**Table:**

S2B—C2B	1.7340 (16)	O2B—Na1A^ii^	2.3521 (13)
S2B—C1B	1.7583 (15)	O2B—H201	0.8609
S2A—C2A	1.7354 (15)	O2B—H202	0.8632
S2A—C1A	1.7578 (15)	N2B—C2B	1.306 (2)
S1B—C1B	1.7211 (16)	N2B—N3B	1.3989 (19)
S1A—C1A	1.7207 (16)	N2A—C2A	1.3071 (19)
Na1B—O2B	2.3433 (13)	N2A—N3A	1.4100 (18)
Na1B—O2A	2.3835 (13)	N3B—C1B	1.304 (2)
Na1B—O1B^i^	2.4576 (17)	O1B—Na1B^i^	2.4576 (17)
Na1B—O1B	2.4619 (16)	O1B—H204	0.8227
Na1B—Na1A	3.2739 (10)	O1B—H203	0.8710
Na1A—O2B^ii^	2.3521 (13)	N3A—C1A	1.3082 (19)
Na1A—O1A	2.3776 (14)	N3A—Na1B^ii^	2.6485 (15)
Na1A—O2A	2.3862 (14)	N3A—Na1A^ii^	2.7735 (15)
Na1A—O1A^iii^	2.5141 (15)	C2A—N1A	1.358 (2)
O2A—H104	0.8538	N1A—H1A1	0.8600
O2A—H103	0.8559	N1A—H1A2	0.8600
O1A—Na1A^iii^	2.5141 (15)	C2B—N1B	1.354 (2)
O1A—H101	0.8545	N1B—H1B1	0.8600
O1A—H102	0.8684	N1B—H1B2	0.8600
			
C2B—S2B—C1B	87.65 (8)	Na1B—Na1A—Na1A^iii^	139.01 (3)
C2A—S2A—C1A	87.70 (7)	O2B^ii^—Na1A—Na1B^ii^	36.27 (3)
O2B—Na1B—O2A	170.14 (5)	O1A—Na1A—Na1B^ii^	119.10 (4)
O2B—Na1B—O1B^i^	93.54 (5)	O2A—Na1A—Na1B^ii^	138.61 (4)
O2A—Na1B—O1B^i^	95.71 (5)	O1A^iii^—Na1A—Na1B^ii^	114.91 (4)
O2B—Na1B—O1B	88.66 (5)	N2A—Na1A—Na1B^ii^	66.59 (3)
O2A—Na1B—O1B	96.17 (5)	N3A^ii^—Na1A—Na1B^ii^	63.54 (3)
O1B^i^—Na1B—O1B	81.01 (6)	Na1B—Na1A—Na1B^ii^	92.07 (2)
O2B—Na1B—N3A^ii^	88.74 (5)	Na1A^iii^—Na1A—Na1B^ii^	128.47 (3)
O2A—Na1B—N3A^ii^	86.31 (5)	Na1B—O2A—Na1A	86.69 (4)
O1B^i^—Na1B—N3A^ii^	99.79 (5)	Na1B—O2A—H104	130.3
O1B—Na1B—N3A^ii^	177.32 (5)	Na1A—O2A—H104	124.2
O2B—Na1B—N2A	88.52 (5)	Na1B—O2A—H103	101.1
O2A—Na1B—N2A	82.33 (4)	Na1A—O2A—H103	104.4
O1B^i^—Na1B—N2A	177.48 (5)	H104—O2A—H103	106.1
O1B—Na1B—N2A	97.60 (5)	Na1A—O1A—Na1A^iii^	93.36 (5)
N3A^ii^—Na1B—N2A	81.70 (5)	Na1A—O1A—H101	129.8
O2B—Na1B—Na1A	123.85 (4)	Na1A^iii^—O1A—H101	98.1
O2A—Na1B—Na1A	46.69 (3)	Na1A—O1A—H102	125.7
O1B^i^—Na1B—Na1A	129.72 (5)	Na1A^iii^—O1A—H102	102.5
O1B—Na1B—Na1A	126.71 (4)	H101—O1A—H102	99.2
N3A^ii^—Na1B—Na1A	54.62 (3)	Na1B—O2B—Na1A^ii^	107.31 (5)
N2A—Na1B—Na1A	49.64 (3)	Na1B—O2B—H201	107.3
O2B—Na1B—Na1B^i^	91.44 (4)	Na1A^ii^—O2B—H201	105.8
O2A—Na1B—Na1B^i^	97.82 (4)	Na1B—O2B—H202	105.6
O1B^i^—Na1B—Na1B^i^	40.55 (4)	Na1A^ii^—O2B—H202	118.2
O1B—Na1B—Na1B^i^	40.46 (4)	H201—O2B—H202	112.2
N3A^ii^—Na1B—Na1B^i^	140.27 (5)	C2B—N2B—N3B	112.60 (13)
N2A—Na1B—Na1B^i^	138.02 (5)	C2A—N2A—N3A	112.15 (12)
Na1A—Na1B—Na1B^i^	144.42 (3)	C2A—N2A—Na1A	126.84 (10)
O2B—Na1B—Na1A^ii^	36.43 (3)	N3A—N2A—Na1A	110.19 (9)
O2A—Na1B—Na1A^ii^	134.54 (4)	C2A—N2A—Na1B	111.62 (10)
O1B^i^—Na1B—Na1A^ii^	117.21 (4)	N3A—N2A—Na1B	114.97 (9)
O1B—Na1B—Na1A^ii^	118.11 (4)	Na1A—N2A—Na1B	76.41 (4)
N3A^ii^—Na1B—Na1A^ii^	59.24 (3)	C1B—N3B—N2B	113.37 (13)
N2A—Na1B—Na1A^ii^	65.29 (3)	Na1B^i^—O1B—Na1B	98.99 (6)
Na1A—Na1B—Na1A^ii^	87.93 (2)	Na1B^i^—O1B—H204	129.2
Na1B^i^—Na1B—Na1A^ii^	127.63 (3)	Na1B—O1B—H204	101.7
O2B^ii^—Na1A—O1A	95.93 (5)	Na1B^i^—O1B—H203	115.5
O2B^ii^—Na1A—O2A	170.82 (5)	Na1B—O1B—H203	112.5
O1A—Na1A—O2A	92.97 (5)	H204—O1B—H203	98.3
O2B^ii^—Na1A—O1A^iii^	87.56 (5)	C1A—N3A—N2A	113.29 (12)
O1A—Na1A—O1A^iii^	86.64 (5)	C1A—N3A—Na1B^ii^	115.21 (10)
O2A—Na1A—O1A^iii^	90.73 (5)	N2A—N3A—Na1B^ii^	123.97 (9)
O2B^ii^—Na1A—N2A	95.52 (5)	C1A—N3A—Na1A^ii^	106.70 (10)
O1A—Na1A—N2A	96.34 (5)	N2A—N3A—Na1A^ii^	116.01 (9)
O2A—Na1A—N2A	85.71 (5)	Na1B^ii^—N3A—Na1A^ii^	74.25 (4)
O1A^iii^—Na1A—N2A	175.46 (5)	N2A—C2A—N1A	123.68 (14)
O2B^ii^—Na1A—N3A^ii^	87.67 (4)	N2A—C2A—S2A	114.17 (11)
O1A—Na1A—N3A^ii^	176.24 (5)	N1A—C2A—S2A	122.15 (12)
O2A—Na1A—N3A^ii^	83.48 (4)	C2A—N1A—H1A1	120.0
O1A^iii^—Na1A—N3A^ii^	94.61 (4)	C2A—N1A—H1A2	120.0
N2A—Na1A—N3A^ii^	82.20 (4)	H1A1—N1A—H1A2	120.0
O2B^ii^—Na1A—Na1B	127.81 (4)	N3B—C1B—S1B	126.07 (12)
O1A—Na1A—Na1B	125.29 (4)	N3B—C1B—S2B	112.60 (12)
O2A—Na1A—Na1B	46.62 (3)	S1B—C1B—S2B	121.34 (9)
O1A^iii^—Na1A—Na1B	121.51 (4)	N2B—C2B—N1B	122.96 (15)
N2A—Na1A—Na1B	53.95 (4)	N2B—C2B—S2B	113.76 (12)
N3A^ii^—Na1A—Na1B	51.13 (3)	N1B—C2B—S2B	123.27 (13)
O2B^ii^—Na1A—Na1A^iii^	92.23 (4)	C2B—N1B—H1B1	120.0
O1A—Na1A—Na1A^iii^	44.83 (4)	C2B—N1B—H1B2	120.0
O2A—Na1A—Na1A^iii^	92.49 (4)	H1B1—N1B—H1B2	120.0
O1A^iii^—Na1A—Na1A^iii^	41.81 (3)	N3A—C1A—S1A	126.40 (12)
N2A—Na1A—Na1A^iii^	141.05 (5)	N3A—C1A—S2A	112.69 (11)
N3A^ii^—Na1A—Na1A^iii^	136.32 (4)	S1A—C1A—S2A	120.89 (9)
			
O2B—Na1B—Na1A—O2B^ii^	13.17 (9)	Na1A—Na1B—O2B—Na1A^ii^	−10.87 (7)
O2A—Na1B—Na1A—O2B^ii^	−170.38 (7)	Na1B^i^—Na1B—O2B—Na1A^ii^	173.99 (4)
O1B^i^—Na1B—Na1A—O2B^ii^	−117.66 (7)	O2B^ii^—Na1A—N2A—C2A	119.25 (13)
O1B—Na1B—Na1A—O2B^ii^	130.87 (7)	O1A—Na1A—N2A—C2A	22.63 (14)
N3A^ii^—Na1B—Na1A—O2B^ii^	−46.25 (6)	O2A—Na1A—N2A—C2A	−69.88 (14)
N2A—Na1B—Na1A—O2B^ii^	65.53 (6)	N3A^ii^—Na1A—N2A—C2A	−153.88 (14)
Na1B^i^—Na1B—Na1A—O2B^ii^	−175.20 (6)	Na1B—Na1A—N2A—C2A	−107.01 (14)
Na1A^ii^—Na1B—Na1A—O2B^ii^	6.74 (5)	Na1A^iii^—Na1A—N2A—C2A	18.77 (17)
O2B—Na1B—Na1A—O1A	−122.01 (6)	Na1B^ii^—Na1A—N2A—C2A	141.65 (14)
O2A—Na1B—Na1A—O1A	54.43 (6)	O2B^ii^—Na1A—N2A—N3A	−21.79 (10)
O1B^i^—Na1B—Na1A—O1A	107.16 (7)	O1A—Na1A—N2A—N3A	−118.40 (10)
O1B—Na1B—Na1A—O1A	−4.31 (8)	O2A—Na1A—N2A—N3A	149.09 (10)
N3A^ii^—Na1B—Na1A—O1A	178.56 (6)	N3A^ii^—Na1A—N2A—N3A	65.09 (10)
N2A—Na1B—Na1A—O1A	−69.65 (6)	Na1B—Na1A—N2A—N3A	111.96 (10)
Na1B^i^—Na1B—Na1A—O1A	49.61 (9)	Na1A^iii^—Na1A—N2A—N3A	−122.26 (9)
Na1A^ii^—Na1B—Na1A—O1A	−128.44 (5)	Na1B^ii^—Na1A—N2A—N3A	0.61 (8)
O2B—Na1B—Na1A—O2A	−176.45 (7)	O2B^ii^—Na1A—N2A—Na1B	−133.74 (4)
O1B^i^—Na1B—Na1A—O2A	52.73 (7)	O1A—Na1A—N2A—Na1B	129.64 (4)
O1B—Na1B—Na1A—O2A	−58.74 (7)	O2A—Na1A—N2A—Na1B	37.13 (4)
N3A^ii^—Na1B—Na1A—O2A	124.13 (6)	N3A^ii^—Na1A—N2A—Na1B	−46.86 (4)
N2A—Na1B—Na1A—O2A	−124.09 (6)	Na1A^iii^—Na1A—N2A—Na1B	125.78 (6)
Na1B^i^—Na1B—Na1A—O2A	−4.82 (7)	Na1B^ii^—Na1A—N2A—Na1B	−111.34 (3)
Na1A^ii^—Na1B—Na1A—O2A	177.12 (5)	O2B—Na1B—N2A—C2A	−96.54 (11)
O2B—Na1B—Na1A—O1A^iii^	127.53 (6)	O2A—Na1B—N2A—C2A	87.15 (11)
O2A—Na1B—Na1A—O1A^iii^	−56.03 (6)	O1B—Na1B—N2A—C2A	−8.10 (11)
O1B^i^—Na1B—Na1A—O1A^iii^	−3.30 (8)	N3A^ii^—Na1B—N2A—C2A	174.51 (11)
O1B—Na1B—Na1A—O1A^iii^	−114.77 (7)	Na1A—Na1B—N2A—C2A	124.59 (11)
N3A^ii^—Na1B—Na1A—O1A^iii^	68.10 (6)	Na1B^i^—Na1B—N2A—C2A	−6.04 (14)
N2A—Na1B—Na1A—O1A^iii^	179.89 (6)	Na1A^ii^—Na1B—N2A—C2A	−125.60 (11)
Na1B^i^—Na1B—Na1A—O1A^iii^	−60.85 (8)	O2B—Na1B—N2A—N3A	32.66 (10)
Na1A^ii^—Na1B—Na1A—O1A^iii^	121.10 (5)	O2A—Na1B—N2A—N3A	−143.65 (10)
O2B—Na1B—Na1A—N2A	−52.36 (6)	O1B—Na1B—N2A—N3A	121.10 (10)
O2A—Na1B—Na1A—N2A	124.09 (6)	N3A^ii^—Na1B—N2A—N3A	−56.29 (11)
O1B^i^—Na1B—Na1A—N2A	176.81 (7)	Na1A—Na1B—N2A—N3A	−106.21 (10)
O1B—Na1B—Na1A—N2A	65.34 (7)	Na1B^i^—Na1B—N2A—N3A	123.16 (10)
N3A^ii^—Na1B—Na1A—N2A	−111.79 (5)	Na1A^ii^—Na1B—N2A—N3A	3.60 (9)
Na1B^i^—Na1B—Na1A—N2A	119.27 (8)	O2B—Na1B—N2A—Na1A	138.86 (4)
Na1A^ii^—Na1B—Na1A—N2A	−58.79 (4)	O2A—Na1B—N2A—Na1A	−37.45 (4)
O2B—Na1B—Na1A—N3A^ii^	59.43 (6)	O1B—Na1B—N2A—Na1A	−132.69 (5)
O2A—Na1B—Na1A—N3A^ii^	−124.13 (6)	N3A^ii^—Na1B—N2A—Na1A	49.92 (4)
O1B^i^—Na1B—Na1A—N3A^ii^	−71.40 (6)	Na1B^i^—Na1B—N2A—Na1A	−130.63 (6)
O1B—Na1B—Na1A—N3A^ii^	177.13 (7)	Na1A^ii^—Na1B—N2A—Na1A	109.80 (3)
N2A—Na1B—Na1A—N3A^ii^	111.79 (5)	C2B—N2B—N3B—C1B	−0.4 (2)
Na1B^i^—Na1B—Na1A—N3A^ii^	−128.95 (8)	O2B—Na1B—O1B—Na1B^i^	−93.80 (5)
Na1A^ii^—Na1B—Na1A—N3A^ii^	52.99 (4)	O2A—Na1B—O1B—Na1B^i^	94.83 (5)
O2B—Na1B—Na1A—Na1A^iii^	178.67 (5)	O1B^i^—Na1B—O1B—Na1B^i^	0.0
O2A—Na1B—Na1A—Na1A^iii^	−4.89 (6)	N2A—Na1B—O1B—Na1B^i^	177.88 (5)
O1B^i^—Na1B—Na1A—Na1A^iii^	47.84 (8)	Na1A—Na1B—O1B—Na1B^i^	133.56 (5)
O1B—Na1B—Na1A—Na1A^iii^	−63.63 (8)	Na1A^ii^—Na1B—O1B—Na1B^i^	−116.13 (5)
N3A^ii^—Na1B—Na1A—Na1A^iii^	119.24 (6)	C2A—N2A—N3A—C1A	0.06 (19)
N2A—Na1B—Na1A—Na1A^iii^	−128.97 (7)	Na1A—N2A—N3A—C1A	147.14 (11)
Na1B^i^—Na1B—Na1A—Na1A^iii^	−9.71 (10)	Na1B—N2A—N3A—C1A	−128.88 (11)
Na1A^ii^—Na1B—Na1A—Na1A^iii^	172.24 (6)	C2A—N2A—N3A—Na1B^ii^	−148.06 (11)
O2B—Na1B—Na1A—Na1B^ii^	6.43 (4)	Na1A—N2A—N3A—Na1B^ii^	−0.97 (13)
O2A—Na1B—Na1A—Na1B^ii^	−177.12 (5)	Na1B—N2A—N3A—Na1B^ii^	83.01 (11)
O1B^i^—Na1B—Na1A—Na1B^ii^	−124.39 (6)	C2A—N2A—N3A—Na1A^ii^	123.98 (12)
O1B—Na1B—Na1A—Na1B^ii^	124.14 (6)	Na1A—N2A—N3A—Na1A^ii^	−88.94 (9)
N3A^ii^—Na1B—Na1A—Na1B^ii^	−52.99 (4)	Na1B—N2A—N3A—Na1A^ii^	−4.96 (12)
N2A—Na1B—Na1A—Na1B^ii^	58.79 (4)	N3A—N2A—C2A—N1A	−179.29 (15)
Na1B^i^—Na1B—Na1A—Na1B^ii^	178.06 (7)	Na1A—N2A—C2A—N1A	40.3 (2)
Na1A^ii^—Na1B—Na1A—Na1B^ii^	0.0	Na1B—N2A—C2A—N1A	−48.62 (19)
O1B^i^—Na1B—O2A—Na1A	−142.04 (5)	N3A—N2A—C2A—S2A	−0.11 (17)
O1B—Na1B—O2A—Na1A	136.43 (5)	Na1A—N2A—C2A—S2A	−140.52 (9)
N3A^ii^—Na1B—O2A—Na1A	−42.56 (4)	Na1B—N2A—C2A—S2A	130.56 (8)
N2A—Na1B—O2A—Na1A	39.55 (4)	C1A—S2A—C2A—N2A	0.09 (13)
Na1B^i^—Na1B—O2A—Na1A	177.17 (4)	C1A—S2A—C2A—N1A	179.29 (15)
Na1A^ii^—Na1B—O2A—Na1A	−4.04 (7)	N2B—N3B—C1B—S1B	−178.64 (12)
O1A—Na1A—O2A—Na1B	−138.33 (5)	N2B—N3B—C1B—S2B	1.37 (18)
O1A^iii^—Na1A—O2A—Na1B	135.00 (4)	C2B—S2B—C1B—N3B	−1.48 (13)
N2A—Na1A—O2A—Na1B	−42.18 (4)	C2B—S2B—C1B—S1B	178.53 (10)
N3A^ii^—Na1A—O2A—Na1B	40.44 (4)	N3B—N2B—C2B—N1B	178.31 (16)
Na1A^iii^—Na1A—O2A—Na1B	176.79 (4)	N3B—N2B—C2B—S2B	−0.78 (19)
Na1B^ii^—Na1A—O2A—Na1B	4.35 (7)	C1B—S2B—C2B—N2B	1.26 (13)
O2B^ii^—Na1A—O1A—Na1A^iii^	87.19 (5)	C1B—S2B—C2B—N1B	−177.82 (16)
O2A—Na1A—O1A—Na1A^iii^	−90.55 (5)	N2A—N3A—C1A—S1A	−178.68 (11)
O1A^iii^—Na1A—O1A—Na1A^iii^	0.0	Na1B^ii^—N3A—C1A—S1A	−27.64 (17)
N2A—Na1A—O1A—Na1A^iii^	−176.56 (5)	Na1A^ii^—N3A—C1A—S1A	52.46 (15)
Na1B—Na1A—O1A—Na1A^iii^	−126.85 (5)	N2A—N3A—C1A—S2A	0.01 (17)
Na1B^ii^—Na1A—O1A—Na1A^iii^	116.75 (4)	Na1B^ii^—N3A—C1A—S2A	151.06 (7)
O1B^i^—Na1B—O2B—Na1A^ii^	133.46 (6)	Na1A^ii^—N3A—C1A—S2A	−128.85 (8)
O1B—Na1B—O2B—Na1A^ii^	−145.64 (5)	C2A—S2A—C1A—N3A	−0.06 (12)
N3A^ii^—Na1B—O2B—Na1A^ii^	33.73 (5)	C2A—S2A—C1A—S1A	178.72 (10)
N2A—Na1B—O2B—Na1A^ii^	−48.00 (5)		

Symmetry codes: (i) −*x*+1, −*y*+1, −*z*+1; (ii) −*x*+2, −*y*+1, −*z*+1; (iii) −*x*+2, −*y*+1, −*z*.

### Hydrogen-bond geometry (Å, °)

**Table:**

*D*—H···*A*	*D*—H	H···*A*	*D*···*A*	*D*—H···*A*
O2A—H104···N3B	0.85	1.99	2.8113 (18)	161
O1A—H102···S1B	0.87	2.39	3.2563 (14)	172
O2B—H201···S1B^iv^	0.86	2.45	3.2962 (12)	169
O2B—H202···N2B^i^	0.86	1.95	2.8024 (18)	170
N1A—H1A2···S1B^v^	0.86	2.57	3.4081 (16)	165
N1B—H1B2···S1A^vi^	0.86	2.43	3.2589 (17)	161

Symmetry codes: (iv) *x*, *y*, *z*+1; (i) −*x*+1, −*y*+1, −*z*+1; (v) *x*, −*y*+3/2, *z*+1/2; (vi) *x*−1, −*y*+3/2, *z*−1/2.
